# Development and External Validation of a Predictive Model of Severe Neonatal Calf Diarrhea in Hanwoo Calves Using Animal, Environmental, and Management Risk Factors

**DOI:** 10.1111/jvim.70238

**Published:** 2025-09-18

**Authors:** Youngjun Kim, Young‐Hwan Lim, Youngwoo Jung, Ji‐Yeong Ku, DoHyeon Yu, Jinho Park

**Affiliations:** ^1^ Department of Veterinary Internal Medicine College of Veterinary Medicine, Jeonbuk National University Iksan Republic of Korea; ^2^ Hanwoo (Korean Indigenous Cattle) Genetic Improvement Center Nonghyup Agribusiness Group Inc. Seosan Republic of Korea; ^3^ Institute of Animal Medicine, College of Veterinary Medicine Gyeongsang National University Jinju Republic of Korea

**Keywords:** critical care, fluid therapy, newborn calves, predicting model

## Abstract

**Background:**

Neonatal calf diarrhea accounts for most pre‐weaned calf losses in Hanwoo cattle. A novel predictive model of severe neonatal calf diarrhea will help veterinarians and farmers prevent disease in calves.

**Hypothesis/Objectives:**

Development and external validation of a simple predictive model for severe neonatal calf diarrhea in Hanwoo cattle.

**Animals:**

Hanwoo calves were used to develop the model (*n* = 3179) and for its external validation (*n* = 1383).

**Methods:**

Retrospective, observational study. The predictive model was developed using logistic regression analysis with data from Hanwoo calves from 2019 to 2022. The model was externally validated using data from Hanwoo calves in 2018 and 2023.

**Results:**

After univariable and multivariable logistic analyses, the month of birth, rainy weather, duration of pregnancy, dam parity, retained fetal membranes, prevalence of neonatal calf diarrhea, induction of parturition, bedding type, and management of failure transfer of passive immunity were selected as predictors, with a sensitivity of 74.1% (95% confidence interval [CI]: 68.9%–78.7%) and specificity of 72.2% (95% CI: 70.6%–73.8%; area under the curve [AUC]: 0.79, 95% CI: 0.766–0.814). In external validation, the accuracy was 83.3% (95% CI: 81.2%–85.2%). Sensitivity and specificity were 60% (95% CI: 50.0%–69.3%) and 85% (95% CI: 82.9%–86.9%), respectively.

**Conclusions and Clinical Importance:**

We have identified predictors for severe neonatal calf diarrhea in Hanwoo calves and have developed a simple, easily calculated scoring prediction model based on these predictors.

**Abbreviations**
CI: confidence interval
FMD: foot and mouth disease
FTPI: failure of transfer of passive immunity
Hanwoo: Korean indigenous cattle
NCD: neonatal calf diarrhea
ROC: receiver operator characteristic

## 1 Introduction

Hanwoo are beef cattle indigenous to Korea that usually are raised in barns with their dams and weaned at 3–4 months of age. As a result, Hanwoo cattle have a high morbidity rate of neonatal calf diarrhea (NCD) [1]. In pre‐weaned calves under 1 month of age, NCD accounts for a substantial proportion of deaths [2]. In the Republic of Korea, NCD has been reported to cause 48.8% and 41.6% of mortality in Hanwoo and dairy calves under 1 month of age, respectively [3, 4].

Infectious diarrhea caused by bovine rotavirus, bovine coronavirus, *Cryptosporidium parvum* , and *Escherichia coli* has been reported to be the most common cause of NCD [5, 6, 7]. Although the causative agents differ, the main pathophysiology of these pathogens is secretory diarrhea caused by toxins or osmotic diarrhea caused by maldigestion and malabsorption as a consequence of villous damage [8, 9, 10, 11]. Secretory and osmotic diarrhea in neonatal calves leads to dehydration, metabolic acidosis, and electrolyte imbalance, which may require treatment with PO or IV fluid therapy [12, 13]. Severe NCD can cause economic losses for Hanwoo cattle producers, including decreased calf growth and increased treatment costs.

Maternal nutrition, dystocia, dam parity, stocking density, bedding, building drainage, building ventilation, colostrum management, vaccination, adverse weather conditions, damp conditions, and herd size have been reported as risk factors for NCD [14, 15, 16, 17, 18, 19]. Management interventions to prevent NCD include decreasing exposure to infectious agents, enhancing specific and nonspecific immunity, ensuring adequate colostrum intake, and promoting farm biosecurity [20, 21].

We aimed to develop a prediction model that could be readily utilized by Hanwoo farmers and veterinarians to predict the likelihood of severe NCD in Hanwoo cattle, considering various factors such as calf characteristics, dam‐related variables, and environmental influences. Whereas existing calf diarrhea prediction models primarily focus on analyzing risk factors for diarrhea, our research prioritized identifying predictors of severe NCD in Hanwoo calves and constructing a simple scoring prediction model, easily calculable by anyone, based on these predictors [22, 23].

## 2 Materials and Methods

### 2.1 Animals

From 2019 to 2022, we registered 3328 calves in a single large Hanwoo farm; 124 calves were removed because of stillbirths and deaths immediately after calving, whereas 25 were removed because of accidental deaths. This left a total of 3179 calves for use in the development of the prediction model. Of the 1440 calves produced in 2018 and 2023, 43 were removed because of stillbirth and 14 were removed because of accidental death, leaving 1383 calves available for external validation. The experimental farm employed a seasonal breeding system, with calving occurring twice annually, from February to April and again from August to October. Parturition took place in individual, separate, loose barns. After delivery, and after a period of 1–2 days, dam‐calf pairs were moved to a communal, loose barn for group rearing. Each loose barn accommodated 45 dam‐calf pairs. The feeding management of the dams and the weaning protocol for the calves were consistently applied annually.

### 2.2 Data Resources

All information in the 2018–2023 dataset was collected from a single large Hanwoo farm. Calves, their dams, and environmental factors were collected by farmers on the farm, and disease diagnosis and treatment information was recorded by a large animal practitioner on the farm. The medical records encompassed individual clinical signs, clinicopathological findings, and medication histories. In the case of deceased calves, necropsies were performed by the farm's attending veterinarian, and the findings were documented in the form of necropsy examination reports. The calving date, dam parity, birth weight, pregnancy duration, difficulty in parturition, retained fetal membranes, induction of parturition, type of bedding (rice straw, chaff), disinfectant, vaccination, failure of transfer of passive immunity (FTPI) management, and supplements for non‐specific immune support (over‐the‐counter) were recorded in the electronic and medical records of the experimental farm. To analyze rainy weather, we used the open‐weather data portal of the Republic of Korea Meteorological Administration (https://data.kma.go.kr). Normal birth weight was defined as 20–45 kg, < 20 kg as underweight, and > 45 kg as overweight. Normal pregnancy duration was defined as an average of 285 days, with 271–299 days defined as pregnancy duration, ≤ 270 days as preterm birth, and ≥ 300 days as overdue birth. Difficulty in parturition was divided into five categories: unassisted, 1–2 person assisted, 3–4 person assisted, machine assisted (obstetrical pulley), and cesarean section. Retained fetal membranes were defined as failure to expel the placenta 24 h after delivery and diagnosed by a veterinarian. The prevalence of NCD was defined as the occurrence of at least 1 day where ≥ 3% of the total calf population in a loose barn, where newborn calves were co‐mingled with their dams and other calves, were affected with mild NCD within 5 days of being moved to the barn. Vaccination against rota‐corona virus (BoviROCO, Green cross veterinary products Inc., Korea) was administered by IM injection once 6 weeks before calving and again 4 weeks before calving in primiparous cows and once 4 weeks before calving in multiparous cows. On the experimental farm, a citric acid‐based disinfectant specifically formulated for foot‐and‐mouth disease (FMD) was employed as a barn disinfectant in 2019. Subsequently, a 2% cresol solution was used from 2020 to 2021, followed by a 5% sodium hypochlorite solution in 2022 (Table 1). For a period of time (Table 1), calves with a serum total protein concentration ≤ 5.5 g/dL at 24–26 h after birth were diagnosed as FTPI and fed colostrum replacer (Good Start, Elanco Inc., USA). Measures were implemented to enhance the dams' maternal potential, such as restricting their movement to assist in calf suckling and prolonging the duration of interaction between calves and their dams. No supplementation, immune enhancer, or egg yolk immunoglobulin was given during this period.

**TABLE 1:** Annual climate conditions, disinfectants, bedding type, and implementation of FTPI management.

Climate	2019			2020			2021			2022			Total average (2019–2022)		
	Average temperature (°C)	Number of rainy days	Precipitation (mm)	Average temperature (°C)	Number of rainy days	Precipitation (mm)	Average temperature (°C)	Number of rainy days	Precipitation	Average temperature (°C)	Number of rainy days	Precipitation	Average temperature (°C)	Number of rainy days	Precipitation (mm)
February	0.4	7	30.2	2.5	10	72.3	2.5	6	9.6	−1.1	5	4.7	1.1	7.0	35.7
March	5.8	6	43.7	6.5	4	23	7.5	7	112.8	6.1	9	72.1	6.5	6.5	46.3
April	11	9	43.9	9.8	5	20.7	12.8	8	110.6	12.3	6	52.2	11.5	7.0	38.9
August	25.9	11	121.1	26.2	18	400	25.2	16	217.8	24.9	16	468.7	25.6	15.3	329.9
September	21.6	10	181.1	20.5	9	257.7	21.9	11	206	21.2	6	165.9	21.3	9.0	201.6
October	15.5	5	81	13.3	3	12.6	15.2	8	55.9	13.7	5	160	14.4	5.3	84.5
Disinfectant	FMD disinfectant			2% cresol						5% hypochlorite					
Bedding type	Chaff			Rice straw											
FTPI management	None			None (51.6%), FTPI management (48.4%)			None			FTPI management					

At the experimental farm, farmers were taught to categorize their calves' feces into four stages based on a calf scoring chart developed by the University of Wisconsin‐Madison School of Veterinary Medicine: score 0‐normal; score 1‐semi‐formed, pasty; score 2‐loose but stays on bedding; and score 3‐watery, slips through bedding. Farmers observed their calves' feces twice a day and referred calves with feces scores of 1, 2, and 3 to a veterinarian. The diagnosis of severe NCD requiring fluid therapy included physical examination findings, such as enophthalmos, skin tent tests, and neurologic examination findings. Blood gas analysis (i‐STAT device and EC8+ cartridge, Abbott Point of Care Inc., Abbott Park, IL, USA) was performed if dehydration was confirmed or if clinical signs of metabolic acidosis were suspected without dehydration. Blood was collected anaerobically from a jugular vein using an arterial blood collection syringe (BD Preset, Becton, Dickinson and Company, UK) and analyzed either directly on the farm or within 30 min of transport to the laboratory. Blood gas analysis was calibrated to 39.5°C. As a result, dehydration ≥ 8% and metabolic acidosis with a venous blood pH ≤ 7.2 accompanied by a decrease in total CO_2_ on blood gas analysis were diagnosed as severe NCD, and IV fluid therapy was administered. Although dehydration (6%) and mild metabolic acidosis (> pH 7.2) were not considered severe NCD, PO fluid therapy was administered as needed. The onset of severe or mild NCD in calves was observed during the first 4 weeks postpartum.

## 3 Statistical Analysis

For the development of a predictive model, we applied multiple logistic regression analysis with NCD as the outcome. In the 2019–2022 dataset, we included variables that have been associated with severe NCD in previous studies. The dataset contains many potential variables, and so we started with a univariable analysis and then performed a multivariable analysis. To select predictors, each variable was subjected to univariable logistic regression analysis with severe NCD as the endpoint, and predictors with a *p* value < 0.25 or thought to play a role in the incidence of NCD were subjected to multivariable logistic regression analysis. Multicollinearity analysis was performed using the variance inflation factor. The final predictor was selected using both forward and backward stepwise selections. Although the final model was determined by statistical significance, statistical and qualitative judgments were considered in the sequential process from initial to final model to avoid missing important predictors regardless of statistical significance. The risk score for severe NCD was quantified as the probability of severe NCD development using the β‐coefficients from the final multivariate logistic regression model, as shown in Equation (1). The discrimination of the final logistic regression prediction model was evaluated using receiver operating characteristic (ROC) curve analysis, with the probability of severe NCD as the independent variable and the occurrence of severe NCD as the dependent variable. A cut‐off value also was determined based on the ROC curve. The calibration of the final model was assessed using a calibration plot, the Hosmer‐Lemeshow test, and the calibration slope.

(1) $$\begin{array}{cc} P=\; & \frac{e^{k}}{1+e^{k}}\;(k=\beta_{0}+\beta_{1}X_{1}+\beta_{2}X_{2}+\ldots +\beta_{n}X_{n},;\\ & X:\text{variable};\beta :\beta -\text{coefficient})\ldots \end{array}$$

Using the provided dataset spanning 2018–2023, we designated the 2019–2022 data as the development set and the 2018 (oldest) and 2023 (newest) data as the validation sets. This constitutes an external validation approach with temporally distinct datasets. The probability of NCD, constructed using the 2019–2022 development data, was validated externally using the 2018 and 2023 datasets. Subsequently, accuracy, sensitivity, and specificity were calculated. *p* values (two‐tailed) < 0.05 were considered significant. All analyses were performed using the SPSS Statistics 29 software package for Windows (SPSS Inc., Chicago, IL, USA) and the R programming language (version 4.3.3).

## 4 Results

### 4.1 Univariable Logistic Regression Analysis

The characteristics of the severe NCD group and normal group calves in the dataset for development of the severe NCD prediction model are summarized in the descriptive statistics table (Table S1). Furthermore, the annual climate conditions, specific management practices, and the prevalence of mild and severe NCD within the development dataset from 2019 to 2022 are summarized in Tables 1 and 2, respectively. The median time to initial occurrence of severe NCD between 2019 and 2022 was 7 days, with an interquartile range of 3 days. An analysis of the maternal influence on severe NCD indicated that first and second parity, retained fetal membranes, and induction of parturition increased the risk of severe NCD. Environmentally, the month of birth (March, April, September, and October) and rainy weather increased the risk of severe NCD. Barns with a > 3% incidence of calf diarrhea had a higher odds ratio for severe NCD. In terms of management, the use of FMD disinfectants (citric acid) increased the risk of disease outbreaks, whereas the use of 5% hypochlorite decreased this risk. Vaccination and FTPI management decreased the risk of developing severe NCD (Table 3).

**TABLE 2:** Annual morbidity and mortality rates of mild and severe neonatal calf diarrhea from 2019 to 2022.

	Mild NCD					Severe NCD		Normal calves		Overall calves				
	Fecal score 1	Fecal **score** 2	Fecal score 3	Total	Death	**Severe** NCD	Death	Normal calves	Death	Female	Male	Freemartin	Total	Death
2019	156	75	47	278	1	109	4	502	6	413	471	5	889	11
	(17.5%)	(8.4%)	(5.3%)	(31.3%)	(0.1%)	(12.3%)	(0.4%)	(56.5%)	(0.7%)	(46.5%)	(53.0%)	(0.6%)	(100.0%)	(1.2%)
2020	159	61	52	272	1	64	0	485	6	385	428	8	821	7
	(19.4%)	(7.4%)	(6.3%)	(33.1%)	(0.1%)	(7.8%)	(0.0%)	(59.1%)	(0.7%)	(46.9%)	(52.1%)	(1.0%)	(100.0%)	(0.9%)
2021	148	36	27	211	0	65	1	502	2	359	414	5	778	3
	(19.0%)	(4.6%)	(3.5%)	(27.1%)	(0.0%)	(8.4%)	(0.1%)	(64.5%)	(0.3%)	(46.1%)	(53.2%)	(0.6%)	(100.0%)	(0.4%)
2022	134	45	54	233	1	63	0	395	6	322	365	4	691	7
	(19.4%)	(6.5%)	(7.8%)	(33.7%)	(0.1%)	(9.1%)	(0.0%)	(57.2%)	(0.9%)	(46.6%)	(52.8%)	(0.6%)	(100.0%)	(1.0%)
Total	597	217	180	994	3	301	5	1884	20	1479	1678	22	3179	28
	(18.8%)	(6.8%)	(5.7%)	(31.3%)	(0.1%)	(9.5%)	(0.2%)	(59.3%)	(0.6%)	(46.5%)	(52.8%)	(0.7%)	(100.0%)	(0.9%)

**TABLE 3:** Univariable logistic regression analysis for sever neonatal calf diarrhea.

Variable	Unadjusted odds ratio	95% CI	*p*
Month of birth			
February	Reference		
March	2.224	1.568–3.155	< 0.0001***
April	4.909	2.666–9.041	< 0.0001***
August	1.052	0.737–1.502	> 0.05
September	1.805	1.232–2.645	< 0.01*
October	3.475	1.832–6.595	< 0.0001***
Birth weight			
Normal (20–45 kg)	Reference		
Low (< 20 kg)	0.667	0.206–2.163	> 0.05
High (> 45 kg)	1.448	0.561–3.738	> 0.05
Pregnancy duration			
Normal	Reference		
Premature	1.394	0.812–2.393	> 0.05
Overdue	1.233	0.916–1.660	> 0.05
Dam parity			
≥ 3	Reference		
1, 2	1.995	1.563–2.547	< 0.0001***
Difficulty in parturition			
Unassisted	Reference		
1–2 person assistant	0.647	0.378–1.108	> 0.05
3–4 person assistant	1.048	0.520–2.111	> 0.05
Using machines	0.518	0.069–3.892	> 0.05
Cesarean section	9.316	0.581–149.367	> 0.05
Rainy weather			
No	Reference		
Yes	5.485	4.164–7.224	< 0.0001***
Retained fetal membranes			
No	Reference		
Yes	5.159	2.463–10.806	< 0.0001***
Twin calves			
No	Reference		
Yes	0.955	0.457–1.994	> 0.05
Prevalence of NCD			
No (< 3%)	Reference		
Yes (> 3%)	3.466	2.298–5.227	< 0.0001***
Induction of parturition			
No	Reference		
Yes	5.831	2.104–16.159	< 0.01**
Disinfectant			
2% Cresol	Reference		
5% Hypochlorite	0.598	0.338–1.059	> 0.05
FMD disinfectant	2.729	2.114–3.524	< 0.0001***
Bedding			
Rice straw	Reference		
Chaff	2.788	2.191–3.548	< 0.0001***
Vaccination			
No	Reference		
Yes	0.346	0.263–0.455	< 0.0001***
FTPI management			
No	Reference		
Yes	0.276	0.183–0.416	< 0.0001***
Supplement			
None	Reference		
Yolk–derived immunoglobulin	1.072	0.809–1.421	> 0.05
Immune enhancer	0.742	0.521–1.056	> 0.05

*Note:* *: *P*<0.01 **: *P*<0.001 ***: *P*<0.0001 These symbols denote the degree of statistical significance.

### 4.2 Development, Discrimination, and Calibration of Prediction Model

**FIGURE 1:**
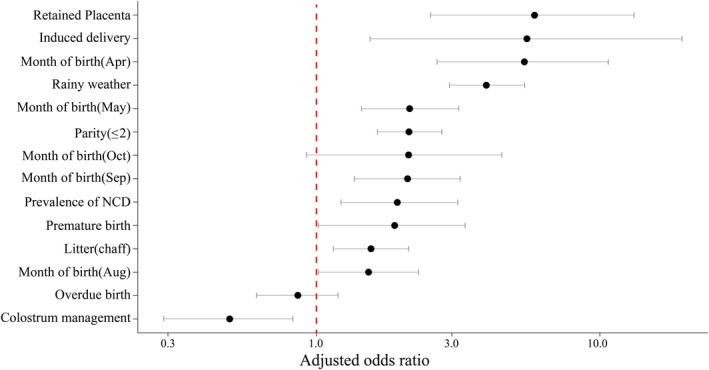
Adjusted odds ratio with 95% confidence interval of predictors from multivariable logistic regression analysis.

After multivariable logistic regression, retained fetal membranes, induction of parturition, dam parity, birth month, gestational period, bedding, and FTPI management were selected as predictors (Table 4, Figure 1). For convenience of the prediction model, all of the predictors were set to the nominal type. Months of birth and pregnancy duration were multi‐categorized, and rainy weather, dam parity, retained fetal membranes, prevalence of NCD, induction of parturition, bedding, and FTPI management were binarily categorized. Retained fetal membranes, induction of parturition, first and second parity, premature birth, and chaff (type of bedding) were selected as predictors of increased risk of severe NCD. In contrast, predictors selected for a decreased risk of severe NCD were over third parity, rice straw (bedding type), overdue birth, and FTPI management (Table 4, Figure 1).

**TABLE 4:** Beta coefficient of multivariable regression analysis used in predictive model for severe neonatal calf diarrhea.

Variables	β‐coefficient	*p*
Intercept	−4.6179	< 0.0001***
Month of birth		
February	Reference	
March	0.7572	< 0.0001***
April	1.6895	< 0.0001***
August	0.4207	< 0.01*
September	0.7394	< 0.0001***
October	0.7472	0.0629
Rainy weather (for 5 days after birth)		
No	Reference	
Yes	1.3794	< 0.0001***
Duration of pregnancy		
Normal	Reference	
Premature	0.6379	< 0.01*
Overdue	−0.1507	**0.37**
Dam parity		
≥ 3	Reference	
1 or 2	0.7538	< 0.0001***
Retained fetal membranes		
No	Reference	
Yes	1.7711	< 0.0001***
Prevalence of neonatal calf diarrhea		
No (< 3%)	Reference	
Yes (≥ 3%)	0.6578	< 0.01*
Induction of parturition		
No	Reference	
Yes	1.7098	< 0.001**
Bedding		
Rice straw	Reference	
Chaff	0.4429	< 0.001**
FTPI management		
No	Reference	
Yes	−0.7050	< 0.001**

*Note:* *: *P*<0.01 **: *P*<0.001 ***: *P*<0.0001 These symbols denote the degree of statistical significance.

**FIGURE 2:**
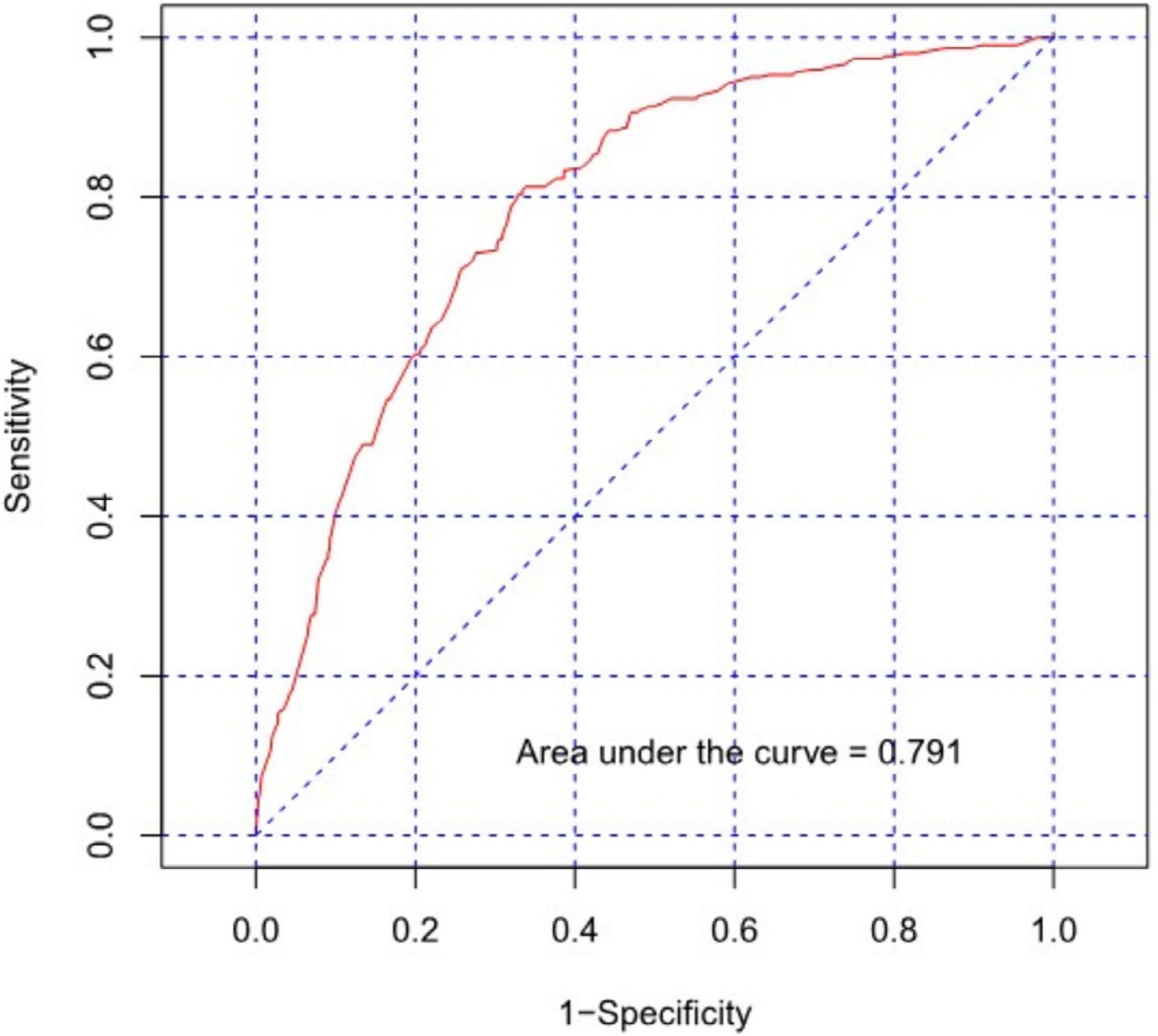
Receiver operator characteristic curves of severe neonatal calf diarrhea using risk score in a multivariable logistic regression analysis model.

**FIGURE 3:**
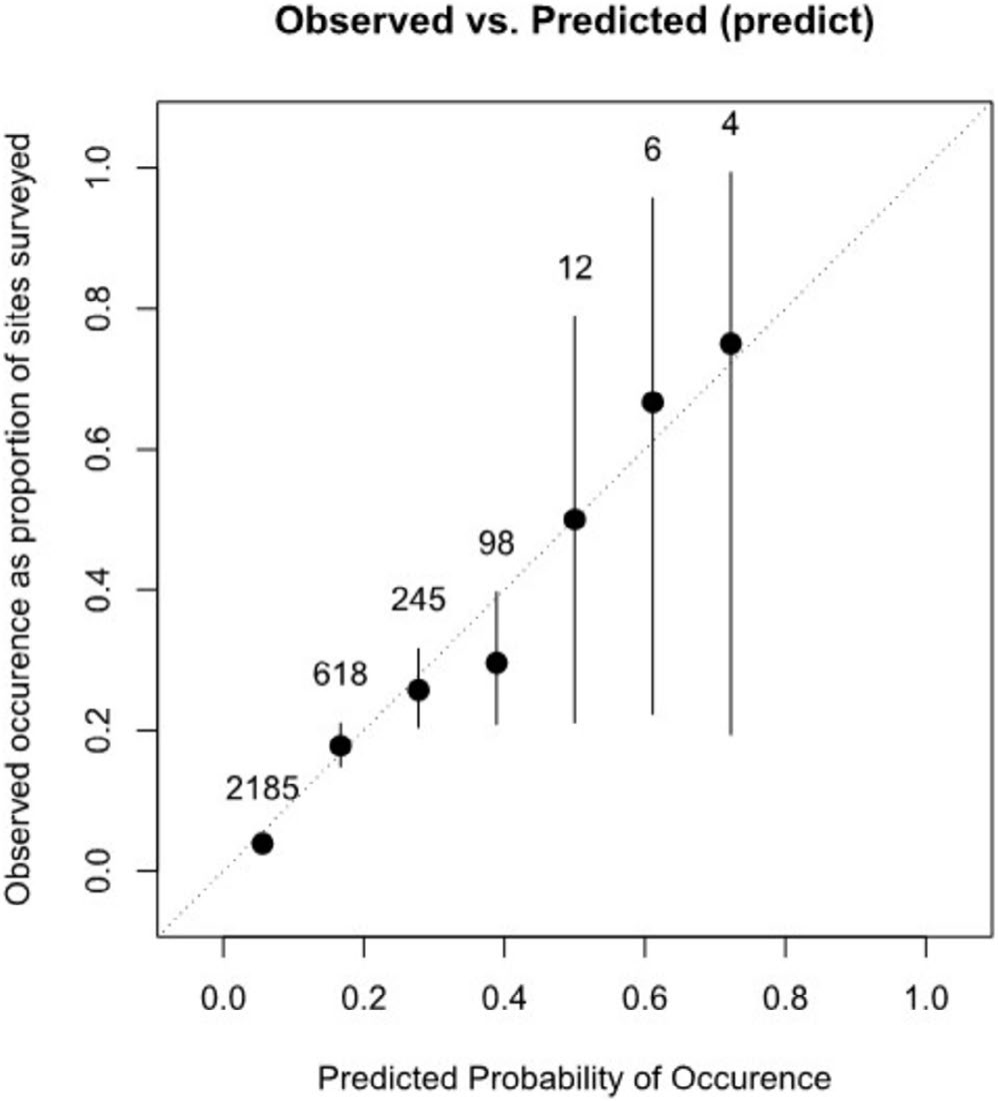
Calibration plot of the severe neonatal calf diarrhea prediction model based on the results of the Hosmer–Lemeshow test.

**FIGURE 4:**
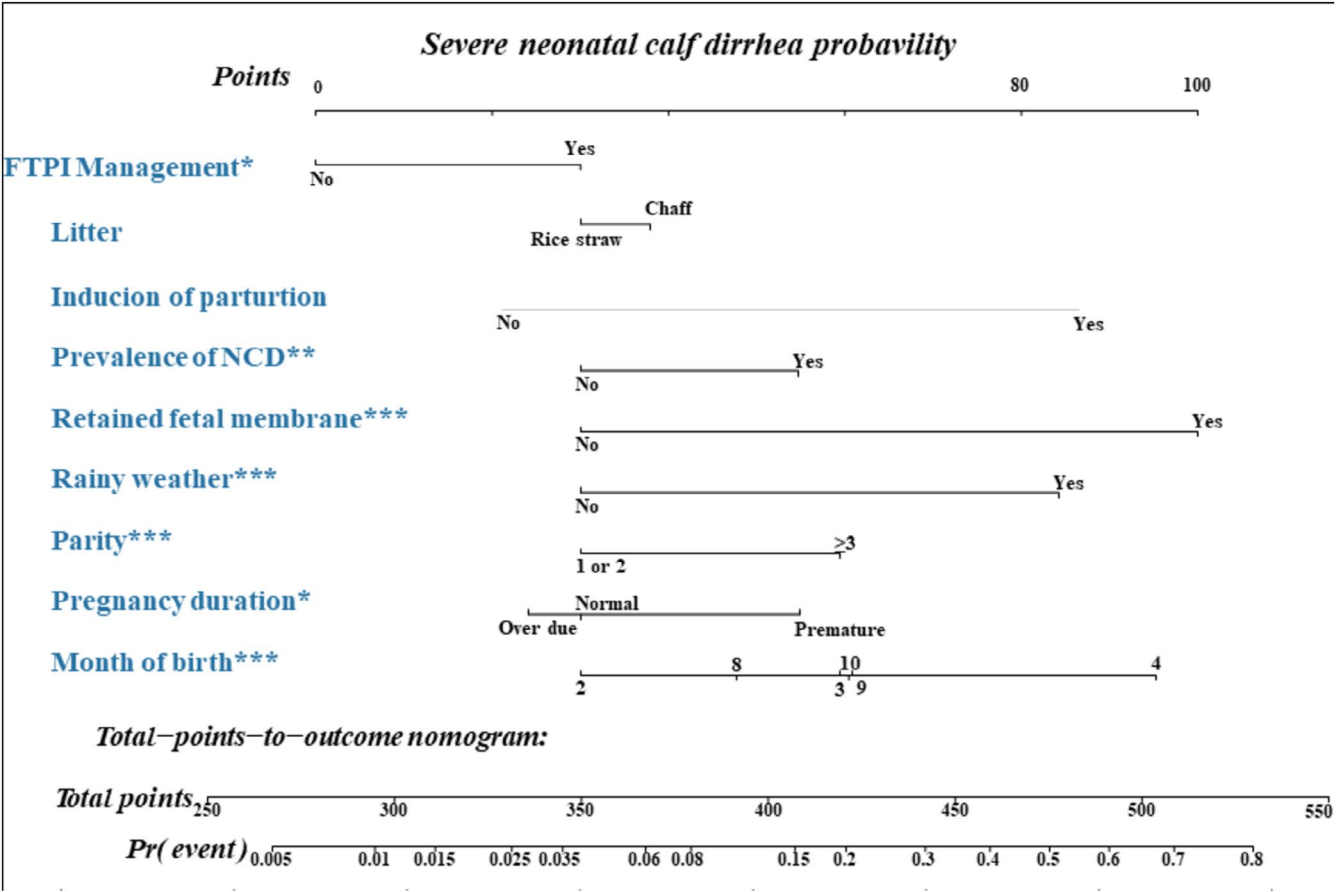
Nomogram to predict probability of severe neonatal calf diarrhea.

We used the β‐coefficients of each predictor to create a risk score variable for the 2019–2022 dataset and performed an ROC curve analysis. The optimal cutoff point was 0.109, the area under the curve was 0.790 (95% confidence interval [CI]: 0.766–0.814), the sensitivity was 0.741 (95% CI: 0.689–0.787), and the specificity was 0.722 (95% CI: 0.706–0.738; Figure 2). The calibration plot and Hosmer‐Lemeshow test indicated that the logistic prediction model was a good fit (*p* = 0.31; Figure 3). The calibration slope was 1.00 (95% CI: 0.88–1.13). A nomogram was created using logistic regression analysis, and the probability of occurrence of severe NCD was calculated (Figure 4). We also developed an application to simplify the calculations for Hanwoo farmers and large animal clinicians (Figure S1).

### 4.3 External Validation of Prediction Model

To ensure robust validation, we used the oldest (2018) and most recent data (2023) for external validation [24]. The datasets from 2018 and 2023 were included in the prediction model. The accuracy was 0.833 (95% CI: 0.812–0.852), with a sensitivity of 0.600 (95% CI: 0.500–0.693) and a specificity of 0.850 (95% CI: 0.829–0.869).

## 5 Discussion

Dam parity, retained fetal membranes, induction of parturition, and duration of pregnancy were identified as dam‐ and calf‐related factors, whereas rainy weather, bedding type, and FTPI management were selected as environmental factors serving as the final predictors. Induction of parturition is a well‐established risk factor for NCD [25, 26, 27]. Some researchers posit that premature delivery resulting from induced parturition can lead to incomplete development of the calf's digestive system, thereby increasing susceptibility to diarrhea‐inducing pathogens [26, 27]. However, in our study, which examined 32 calves born via artificial induction, 11 cases of severe NCD were observed. Of these, 72.7% were associated with normal gestational periods, whereas 9.1% and 18.2% were linked to premature and delayed deliveries, respectively. These findings suggest that, in our observations, NCD is potentially more closely related to decreased colostrum production in dams as a consequence of parturition induction and increased stress in both dams and calves caused by exogenous cortisol, rather than to gastrointestinal immaturity resulting from premature birth [25, 26, 27, 28, 29]. This cascade of events may contribute to the weakening of the calf's immune system, thereby influencing the incidence of NCD.

Twenty‐four calves were born to cows that had experienced retained fetal membranes; of these, eight calves developed severe NCD. Notably, 75% of the dams of these eight calves had received antibiotic treatment for ≥ 5 days. Conversely, only 56.3% of dams of the 16 calves that did not develop severe NCD received antibiotic treatment for a duration of 1–2 days. Cows experiencing more severe retained placenta tended to spend more time lying down than standing in the barn. Consequently, this led to decreased suckling time for calves and decreased maternal care. Considering the aforementioned results collectively, our study suggests that retained fetal membranes may act as a predictive factor in increasing the incidence of severe NCD. Studies have reported fetal membranes retention rates > 75% in cows in which parturition was artificially induced 1–2 weeks before the expected due date [30, 31]. In contrast, induction of parturition several days before or on the expected due date has been associated with placental retention rates ranging from 10% to 50% [30, 31]. The absence of a significant association between induced parturition and retained fetal membranes in our study is likely attributable to the practice of inducing parturition after the expected due date on the experimental farm.

Calves born to heifers have a 3.9 times higher incidence of NCD than calves born to cows that have already calved [15, 17]. This observation is reportedly a consequence of heifers having lower milk yield, colostrum quality, and maternal potential [15]. Premature births have been reported to cause increased mortality and poor growth in calves [32, 33]. In our study, preterm birth was selected as a predictor of severe NCD in the final model. Prematurely born Hanwoo calves tend to be weak and spend considerable time lying down, which is thought to increase their contact time with floor contaminants and contribute to the development of NCD. Premature, weak calves also are less likely to ingest adequate colostrum [34].

Dystocia is known to be a risk factor for NCD [35, 36]. Dystocia affects the suckling ability of calves, decreases colostrum intake, and increases morbidity [20]. It is also known that cows that have experienced dystocia tend to refuse to suckle their calves [37, 38]. We performed univariable and multivariable logistic regression analyses of severe NCD with calving difficulty at four levels, but calving difficulty was not found to be a significant predictor of severe NCD. This finding is thought to be related to the fact that the objective of the previous study was the morbidity of NCD, whereas the objective of our study was the severity of NCD.

Moist conditions or standing rainwater can cause calf diarrhea, particularly if > 5% of the nursing area is affected [21]. From 2019 to 2022, the average number of rainy days (and average rainfall) in August and September was 15.3 days (329.9 mm) and 9 days (201.6 mm), respectively. In contrast, the average number of rainy days in February from 2019 to 2022 was 7 days, with an average rainfall of 35.7 mm. Considering these observations, the high rainfall and number of rainy days in August and September may have contributed to an increased incidence of severe NCD. Whereas the average number of rainy days (and average rainfall) in March and April from 2019 to 2022 was similar at 6.5 days (46.3 mm) and 7 days (38.9 mm) respectively, the increased incidence of severe NCD appears to be associated with the higher prevalence of NCD in March and April compared with February. Furthermore, considering that the experimental farm practices seasonal breeding with both spring and autumn calving, the final months of each calving season (April and October) had a higher incidence of severe NCD compared with February, likely representing the final stages of the calving period. This observation is consistent with previous research indicating an increased incidence of calf diarrhea as the calving season progresses [16, 17].

The cattle sheds at the experimental farm initially were disinfected using water and soap to physically remove contaminants. Subsequently, in 2019, a commercial FMD disinfectant, a compound formulation of benzalkonium chloride and citric acid, was used. From 2020 to 2021, a 2% cresol solution was used, followed by a 5% sodium hypochlorite solution in 2022. Univariable analysis indicated that the use of the FMD disinfectant, compared with the 2% cresol solution, was associated with an increased incidence of severe NCD. This observation aligns with previous studies indicating the efficacy of cresol solution against Gram‐negative bacteria and non‐enveloped viruses, such as rotavirus, as well as its disinfectant properties against cryptosporidium [39, 40, 41, 42]. However, the observed decrease in the incidence of severe NCD with the use of 5% sodium hypochlorite, which lacks documented disinfectant activity against cryptosporidium, contradicts previous research [20, 39, 40, 41, 42]. Furthermore, the exclusion of disinfectant application as a final predictor in multivariable logistic regression suggests that its use was relatively less influential compared with other factors.

Hanwoo farmers primarily used rice straw, chaff, and sawdust as bedding materials. Our study analyzed chaff and rice straw but not sawdust. Rice straw and chaff, as organic bedding materials, can facilitate bacterial proliferation because of increased moisture levels [43]. In our experimental farm, we used both rice straw and chaff as bedding for pre‐weaned calves. The results indicated that the use of chaff, compared with rice straw, significantly increased the incidence of severe NCD. This observation aligns with previous research demonstrating that chaff, relative to rice straw, increases both the cleanness score and fecal fluidity score of barn floors [44, 45]. The increased moisture retention of chaff compared with rice straw, coupled with its finer particle size resulting in prolonged contact with calves, likely facilitated pathogen transmission [46]. Calves spend much of their time lying down, which can lead to intentional or unintentional ingestion of bedding [34]. Abomasum impaction by foreign bodies such as hairballs and hay in the digestive system has been reported in Hanwoo calves [47]. In our study, most dead calves with severe NCD had undigested chaff in their abomasum.

The prevalence of NCD increased the risk of severe NCD, but with a smaller odds ratio than predicted. This finding suggests that the transmission of NCD‐causing agents to calves is not solely attributable to other calves. In our study, primiparous cows constituted > 24% of each calving group. Previous research has identified a primiparous cow ratio > 20% as a risk factor for NCD [21]. Furthermore, calves born to heifers shedding rotavirus and bovine coronavirus are reportedly more likely to develop NCD compared with those born to multiparous carrier cows. We hypothesize that this factor contributed to the lower than expected adjusted odds ratio observed for NCD prevalence.

Unless they develop an intrauterine infection, newborn calves are immunologically naïve. In calves, ingestion of colostrum within 24–36 h of birth establishes passive immunity for 3–5 weeks before adaptive immunity develops [48, 49]. Failure of transfer of passive immunity caused by inadequate colostrum intake has been reported to increase calf mortality [50, 51]. In our study, FTPI management was selected as a predictor in the final model. In addition to promptly administering colostrum replacer to calves diagnosed with FTPI, efforts were made to enhance the maternal care abilities of the dams. These efforts included restraining the dams to facilitate nursing and increasing the duration of interaction between the calves and their dams. Notably, the proportion of primiparous cows on the experimental farm remained consistently high, at 26.2% in 2019, 24.5% in 2020, 24.8% in 2021, and 26.1% in 2022. Given that primiparous cows generally exhibit decreased maternal care abilities compared with multiparous cows, the active intervention of farm personnel to improve these abilities may have contributed to the enhanced management of FTPI, potentially leading to a decrease in the incidence of severe NCD. In our study, rotavirus and coronavirus vaccinations were significant in univariable regression but were not the final predictors in multivariable regression. This result is because our study focused on the severity rather than the morbidity of NCD, and it is believed that the coronavirus strain used to vaccinate Hanwoo cows is different from the coronavirus strain actually causing the outbreak in Hanwoo calves [52, 53]. We agree that vaccines are one way to prevent NCD but they are not a substitute for appropriate management [54]. In addition, many Hanwoo farmers provide supplementation for newborn calves. In our study, two supplementation factors, immune enhancer and yolk‐derived immunoglobulin, were analyzed, but no significant results were found.

Our study had two major limitations. First, it was limited by the diversity of field conditions. Known risk factors, such as herd size, housing type, barn ventilation, and whether the cows and heifers lived together, were not selected as predictors. In addition, the fact that the study was carried out on only one large farm is a major limitation. We have endeavored to derive predictive variables by analyzing the commonalities between data from an experimental farm and typical Hanwoo farms. Fortunately, no unique feeding management practices that would differentiate the experimental farm from other farms were identified in the experimental farm's data. Second, the lack of serum biochemical profiles prevented the creation of a clinical model. However, we were able to improve the accuracy of the prediction model using accurate information recorded from a large single farm, and it is important to create a prediction model that can be easily used by livestock owners using known risk factors as predictors.

## 6 Conclusion

In summary, univariable and multivariable logistic regression analyses were performed to elucidate dam and calf risk factors, including birth weight, twin calves, dystocia, dam parity, retained fetal membranes, induction of parturition, and duration of pregnancy; environmental risk factors, such as nursing area, month of birth, prevalence of NCD, disinfectant, type of bedding, and rainy weather; and management risk factors, such as vaccination and FTPI management. Predictors of increased incidence of severe NCD in Hanwoo calves were retained placenta, induced delivery, rainy weather, month of birth (April, May, October, September, August), parity (≤ 2), prevalence of NCD, premature birth, litter (chaff), and predictors of decreased incidence of severe NCD were overdue birth and FTPI management.

## Disclosure

Authors declare no off‐label use of antimicrobials.

## Ethics Statement

Approved by the Institutional Animal Care and Use Committee (IACUC) of the National Institute of Animal Science, Republic of Korea (JBNU IACUC no. NON2023‐123). Authors declare human ethics approval was not needed.

## Conflicts of Interest

The authors declare no conflicts of interest.

## Supporting information


**Figure S1:** Application designed to predict severe neonatal calf diarrhea in Hanwoo farmers and large‐animal clinicians.

**Data S1:** Supporting Information.
