# Supplementary figures and images for: Development and External Validation of a Predictive Model of Severe Neonatal Calf Diarrhea in Hanwoo Calves Using Animal, Environmental, and Management Risk Factors
Source: J Vet Intern Med. 2025 Sep 18;39(5):e70238. doi: 10.1111/jvim.70238 (PMC12445427; doi:10.1111/jvim.70238)

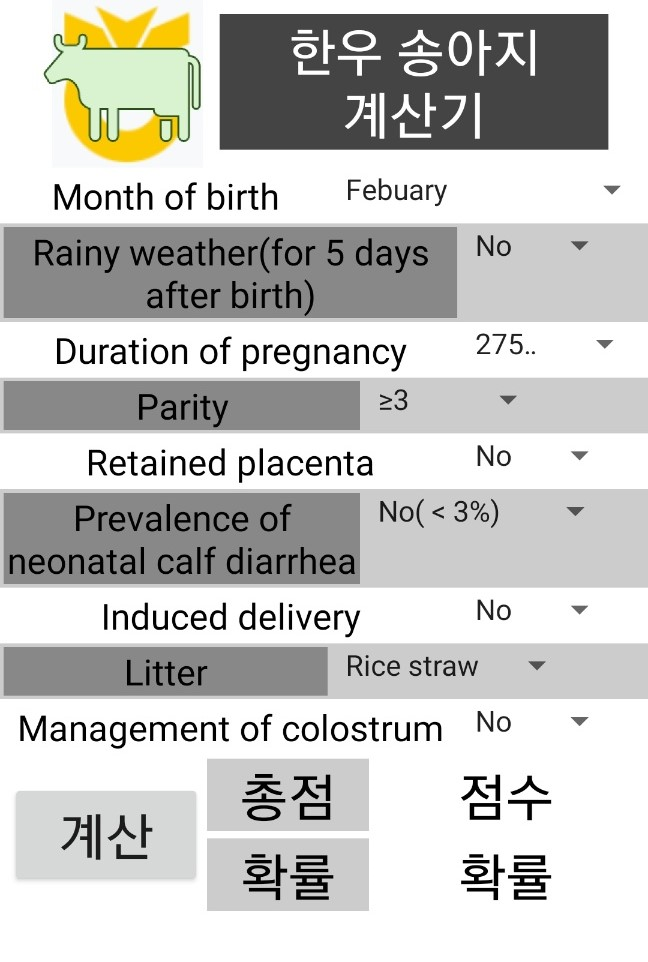

Supplement: Supplementary file 1 — Figure S1: Application designed to predict severe neonatal calf diarrhea in Hanwoo farmers and large‐animal clinicians. [file JVIM-39-e70238-s001.tif]
