# Supplementary material for: Development and External Validation of a Predictive Model of Severe Neonatal Calf Diarrhea in Hanwoo Calves Using Animal, Environmental, and Management Risk Factors
Source: J Vet Intern Med. 2025 Sep 18;39(5):e70238. doi: 10.1111/jvim.70238 (PMC12445427; doi:10.1111/jvim.70238)
Supplement: Supplementary file 2 — Data S1: Supporting Information. [file JVIM-39-e70238-s002.docx]

Supplementary table. Characteristics of the dataset (2019-2022) for the development of a severe NCD prediction model.

|  | Severe NCD | Normal | Total |
| --- | --- | --- | --- |
|  | (N=301) | (N=2878) | (N=3179) |
| Month of birth |  |  |  |
| February | 60 (19.9%) | 850 (29.5%) | 910 (28.6%) |
| March | 83 (27.6%) | 528 (18.3%) | 611 (19.2%) |
| April | 17 (5.6%) | 49 (1.7%) | 66 (2.1%) |
| August | 71 (23.6%) | 955 (33.2%) | 1026 (32.2%) |
| September | 56 (18.6%) | 439 (15.3%) | 495 (15.6%) |
| October | 14 (4.7%) | 57 (2.0%) | 71 (2.2%) |
| Birth weight |  |  |  |
| Normal | 293 (97.3%) | 2802 (97.4%) | 3095 (97.4%) |
| Low | 3 (1.0%) | 43 (1.5%) | 46 (1.4%) |
| High | 5 (1.7%) | 33 (1.1%) | 38 (1.2%) |
| Pregnancy duration |  |  |  |
| Normal | 223 (74.1%) | 2253 (78.3%) | 2476 (77.9%) |
| Premature | 16 (5.3%) | 117 (4.1%) | 133 (4.2%) |
| Overdue | 62 (20.6%) | 508 (17.7%) | 570 (17.9%) |
| Dam parity |  |  |  |
| Over third parity | 114 (37.9%) | 1580 (54.9%) | 1694 (53.3%) |
| First, Second parity | 187 (62.1%) | 1298 (45.1%) | 1485 (46.7%) |
| Difficulty in parturition |  |  |  |
| Unassisted | 275 (91.4%) | 2563 (89.1%) | 2838 (89.3%) |
| 1-2 person assistant | 15 (5.0%) | 216 (7.5%) | 231 (7.3%) |
| 3-4 person assistant | 9 (3.0%) | 80 (2.8%) | 89 (2.8%) |
| Using machines | 1 (0.3%) | 18 (0.6%) | 19 (0.6%) |
| Cesarean section | 1 (0.3%) | 1 (0.0%) | 2 (0.1%) |
| Rainy weather |  |  |  |
| No | 72 (23.9%) | 1822 (63.3%) | 1894 (59.6%) |
| Yes | 229 (76.1%) | 1056 (36.7%) | 1285 (40.4%) |
| Retained fetal **membranes** |  |  |  |
| No | 290 (96.3%) | 2857 (99.3%) | 3147 (99.0%) |
| Yes | 11 (3.7%) | 21 (0.7%) | 32 (1.0%) |
| Twin calves |  |  |  |
| No | 293 (97.3%) | 2797 (97.2%) | 3090 (97.2%) |
| Yes | 8 (2.7%) | 81 (2.8%) | 89 (2.8%) |
| Prevalence of NCD |  |  |  |
| No (<3%) | 26 (8.6%) | 711 (24.7%) | 737 (23.2%) |
| Yes (>3%) | 275 (91.4%) | 2167 (75.3%) | 2442 (76.8%) |
| Induction of parturition |  |  |  |
| No | 294 (98.0%) | 2859 (99.7%) | 3153 (99.5%) |
| Yes | 6 (2.0%) | 10 (0.3%) | 16 (0.5%) |
| Disinfectant |  |  |  |
| 2% Cresol | 100 (33.2%) | 1499 (52.1%) | 1599 (50.3%) |
| 5% Hypochlorite | 187 (62.1%) | 1028 (35.7%) | 1215 (38.2%) |
| FMD disinfectant | 14 (4.7%) | 351 (12.2%) | 365 (11.5%) |
| Bedding |  |  |  |
| Rice straw | 153 (50.8%) | 2136 (74.2%) | 2289 (72.0%) |
| Chaff | 148 (49.2%) | 742 (25.8%) | 890 (28.0%) |
| Vaccination |  |  |  |
| No | 86 (28.6%) | 350 (12.2%) | 436 (13.7%) |
| Yes | 215 (71.4%) | 2528 (87.9%) | 2743 (86.3%) |
| FTPI management |  |  |  |
| No | 275 (91.4%) | 2143 (74.5%) | 2418 (76.1%) |
| Yes | 26 (8.6%) | 735 (25.5%) | 761 (23.9%) |
| Supplement |  |  |  |
| None | 80 (26.6%) | 742 (25.8%) | 822 (25.9%) |
| Yolk–derived immunoglobulin | 163 (54.2%) | 1411 (49.0%) | 1574 (49.5%) |
| Immune enhancer | 58 (19.3%) | 725 (25.2%) | 783 (24.6%) |
